# Phosphatidylserine exposure and plasma membrane perforation as ferroptotic signatures for in vivo imaging

**DOI:** 10.1038/s44303-025-00110-1

**Published:** 2025-10-06

**Authors:** Ali Yasin Sonay, Elana Apfelbaum, Benedict Edward Mc Larney, Jan Grimm

**Affiliations:** 1 Molecular Pharmacology Program, Memorial Sloan Kettering Cancer Center, New York, NY USA; 2 Pharmacology Program, Weill Cornell Medical College, New York, NY USA; 3 Department of Radiology, Memorial Sloan Kettering Cancer Center, New York, NY USA; 4 Department of Radiology, Weill Cornell Medical Center, New York, NY USA

**Keywords:** Biological techniques, Cancer

## Abstract

Ferroptosis emerged as a cell death modality against cancer, but there are currently no available biomarkers for imaging ferroptosis-based therapies. To address this, we evaluated phosphatidylserine exposure and perforation of lipid membranes during ferroptosis to explore potential targeting opportunities. We demonstrated that nano-sized gaps at late stage ferroptosis can serve as entry points for dyes that can bind to intracellular structures. These changes were accompanied with cellular signaling components similar to platelet activation, with phosphatidylserine exposure on the cell surface as a potential target for imaging programed cell death, including ferroptosis. We employed a novel tumor-seeking dye CJ215 that can also label apoptotic cells and showed that CJ215 accumulates in ferroptotic cells both in vitro and in vivo by binding to phosphatidylserine, which can be prevented with ferroptosis inhibition. Since phosphatidylserine exposure also occurs during apoptosis, CJ215 can monitor both apoptosis and ferroptosis-based therapies.

## Introduction

Ferroptosis is a more recently discovered cell death mechanism that is caused by disruption in cellular redox regulations and ultimately results in excess lipid peroxide accumulation in cellular membranes with subsequent membrane instability and disruptions leading to cell death^1^. Lipid peroxidation is thought to occur mostly through the Fenton reaction between hydrogen peroxide and intracellular ferrous iron, generating hydroxyl radicals that oxidize the polyunsaturated fatty acids in the membrane^2^. GPX4 is a crucial enzyme that can reduce these lipid peroxides, so either direct inhibition of GPX4^3^ or depletion of its substrate Glutathione (GSH)^4–6^ can lead to ferroptosis. Over the years, several ferroptosis inducers have been discovered starting with the System Xc- (cystine uptake) inhibitor erastin^7^, GPX4 inhibitor RSL3^8^, GSH synthesis inhibitor BSO^9^ or clinically approved iron oxide nanoparticles^10^ to name a few. It is widely accepted that significant changes in oxidized lipid species occur during (and are a hallmark of) ferroptosis, which are typically characterized by lipidomic approaches such as measuring lipid peroxidation^11^. Beyond the changes in lipid content, lipids’ localization and membrane integrity are essential factors that maintain membrane homeostasis^12^. This homeostasis emphasizes the boundaries between inside and outside of a cell that in turn are responsible for compartmentalization of cellular processes to control pH, ion levels, membrane potential, metabolites, proteins and enzymatic processes^13^. Dysfunctional antioxidant pathways and lipid peroxidation in the membrane initiate signaling cascades that contribute to ferroptosis, but while intense focus has been given to the lipid species that define ferroptotic cell states^14^, little attention is given to the nature of the membrane damage and lipid exposure during the course of late ferroptosis. In this study, we have investigated the causes and characteristics of nano-sized gaps on the membrane as the cells undergo ferroptosis. Furthermore, we took advantage of these membrane changes to employ a new labeling strategy for in vivo monitoring of ferroptosis-based therapy.

## Results

### Shared components between platelet activation and ferroptosis

**Fig. 1:**
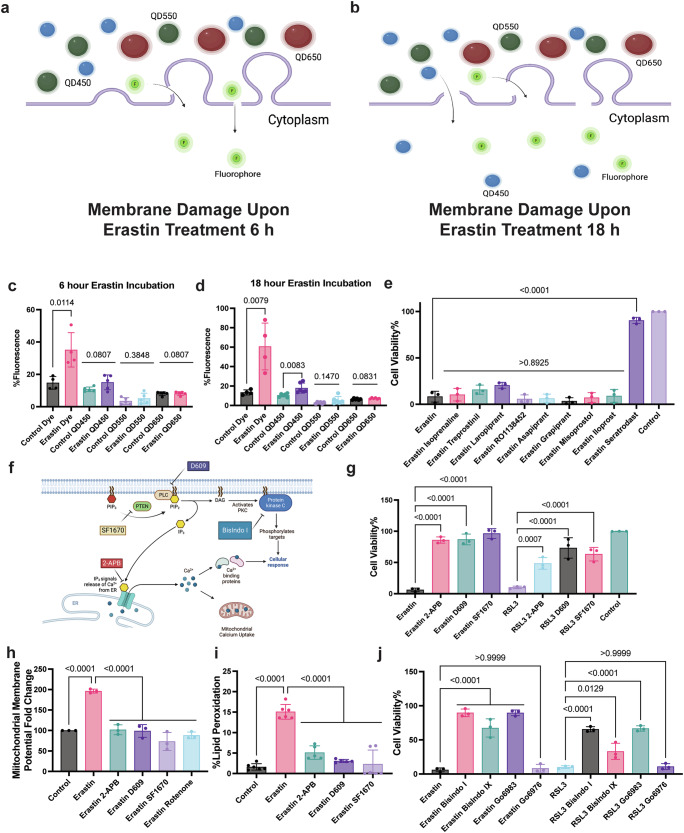
Ferroptosis leads to loss of membrane integrity and shares similar components with platelet activation in MDA-MB-435 cells. **a** Figure showing indicating loss of cellular membrane integrity showing nano-sized gaps less than 5 nm only allowing small molecule dye uptake (done in Biorender). **b** Figure demonstrating loss of cellular membrane integrity showing nano-sized gaps less than 10 nm only allowing dye and QD450 uptake, done in Biorender. **c** Cell membrane penetration of different sizes of quantum dots and DAPI upon erastin treatment for 6 h showing dye uptake but not quantum dot uptake**, d** Cell membrane penetration of different sizes of quantum dots and DAPI upon erastin treatment for 18 h showing increased uptake of DAPI and 5-8 nm quantum dots with 450 nm excitation. **e** Effect of prostaglandin and thromboxane modulators on erastin-induced cytotoxicity showing platelet activation inhibitor seratrodast rescues the cells from ferroptosis. **f** Figure showing key components of platelet activation, where inhibitors of each component are shown in rectangular boxes, done in Biorender. **g** IP_3_R, PLC and PTEN inhibitors 2-APB (2 μM), D609 (5 μM), SF1670 (0.2 μM), respectively, rescue the cells from both erastin and RSL3-induced ferroptosis. **h** Erastin induced increases mitochondrial membrane potential measured with JC10 dye which can be inhibited with IP_3_R, PLC, PTEN inhibitors as well as Electron Transport Chain inhibitor Rotenone (1 μM) **i** IP_3_R, PLC, PTEN inhibitors rescue the cells from erastin-induced lipid peroxidation. **j** Role of different PKC inhibitors and their effect on erastin and RSL3-induced ferroptosis. For cytotoxicity assays *n* = 3 biological replicates, for the lipid peroxidation and mitochondrial membrane potential assays *n* = 6 pooled from *n* = 3 independent experiments. Mean ± s.d. *p* values are reported above the lines (Ordinary one-way ANOVA with Tukey’s multiple comparisons).

Ferroptosis leads to characteristic membrane swelling with blebbing and eventual rupture^15^, but the timescales for these events depend on the cell type, as well as the chosen ferroptosis inducer, and therefore vary widely. To derive a more universal ferroptosis framework, we looked at early-stage (within 6 h of treatment) and late-stage (between 12–18 h post-treatment) erastin-induced ferroptosis. To date, membrane integrity during ferroptosis has been analyzed via cell-impermeable dyes that were typically used for apoptosis assays^16^. While this approach reveals membrane damage with high sensitivity, it does not report the size of any generated membrane disruptions during cell death. To overcome this, we employed a 1 nm sized organic dye (DAPI) as well as water soluble quantum dots with size distributions around 5, 10, and 15 nm, respectively (Supplementary Fig. 1a). Since increasing quantum dot size leads to their red shifted emission spectra, we were able to distinguish passive cellular uptake of different sizes^17^ using flow cytometry during early and late ferroptosis stages (Fig. 1a, b). Our results revealed that during early ferroptosis, membrane damage only permitted the uptake of the 1 nm sized organic dye (Fig. 1c), whereas during late stage ferroptosis the 5 nm sized QD450 were also able to penetrate the damaged membrane, but larger quantum dots still were not able to (Fig. 1d). Previous studies discussed the signaling aspect of lipid peroxide species and their effect on cell rupture and immune cell activation^18^. We investigated whether membrane swelling and integrity disruption in ferroptosis leads to release of intracellular components to the surrounding cells similar to immunogenic cell deaths such as pyroptosis^19^. We tested different adrenergic, prostaglandin and thromboxane receptor modulators^20^ which are involved in immunogenic responses (Supplementary Fig. 2a) to see their effect on and possible role in cells undergoing ferroptosis. We showed that the thromboxane A2 (TXA_2_) receptor inhibitor seratrodast rescues the cells from erastin and RSL3 induced ferroptosis whereas other modulators did not greatly affect ferroptosis onset (Fig. 1e, Supplementary Fig. 2b), Previous studies also showed that inhibition of thromboxane A2 receptor, a G-protein coupled receptor involved in platelet activation, inhibits neuronal ferroptosis by decreasing lipid peroxidation and inhibiting JNK phosphorylation^21^. We reasoned that there could be shared components of the ferroptosis and platelet activation pathways, such as PIP_2_-PLC-IP_3_ receptor-PKC axis^22^ (Fig. 1f) since seratrodast is a platelet activation inhibitor and platelet activation is also accompanied by similar membrane swelling.

One of the key events during platelet activation is the release of calcium from the endoplasmic reticulum using IP_3_ receptors as calcium channels. Our results revealed that IP_3_ receptor inhibitor 2-APB led to the inhibition of both erastin and RSL3-induced ferroptosis. Therefore, we tested whether IP_3_ signaling molecule synthesis also influences ferroptosis. Given that IP_3_ is produced from PIP_2_ signaling molecules via Phospholipase C (PLC), we tested whether inhibition of PLC activity directly with D609 or downstream with 2-APB would rescue from ferroptosis in MDA-MB-435 cells (Fig. 1f, g). Furthermore, PIP_2_ levels are tightly regulated by the balance of IP_3_ kinase (which converts PIP_2_ to PIP_3_) and PTEN, which converts PIP_3_ to PIP_2_. We reasoned that the inhibition of PTEN with SF-1670 would lead to a depletion of the PIP_2_ pool and would, thereby, prevent cells from undergoing ferroptosis. Our results showed both PLC inhibitor D609, PTEN inhibitor SF-1670 rescue the cells from both erastin and RSL3-induced ferroptosis (Fig. 1g). To confirm that the observed effect was not cell-specific, we tested these inhibitors in HT1080 cells and showed again that PLC and PTEN inhibitors rescued the cells from erastin and RSL3, while the IP_3_ receptor inhibition only prevents erastin-induced ferroptosis (Supplementary Fig. 3a), which may indicate that RSL3-induced ferroptosis does not rely on calcium signaling in HT-1080 cells. Previous studies also showed erastin treatment led to an increase in mitochondrial membrane potential which is inhibited by electron transport chain inhibitor rotenone^23^, and we demonstrated IP_3_ receptor, PLC and PTEN inhibitors also decrease the mitochondrial membrane potential similar to rotenone (Fig. 1h). Erastin-induced lipid peroxidation can also be prevented using IP_3_ receptor, PLC and PTEN inhibitors (Fig. 1i). Upon PLC activity, PIP_2_ molecules are hydrolyzed to IP_3_ and DAG. As a result, both and IP_3_ receptor-induced calcium release and DAG activity lead to Protein Kinase C (PKC) activation. Yet, there are different classes of PKCs and each has diverse functions depending on the cell type and environmental conditions. Given the redundancy of PKC activation, it is hard to distinguish which PKC isoform is responsible for ferroptosis; therefore, we curated a small PKC inhibitor library to test multiple PKC inhibitors (see Supplementary Fig. 3b) to distinguish whether we can identify the responsible PKC types. We observed that both erastin- and RSL3-induced ferroptosis could be rescued by the PKC inhibitors BisIndolmaleimide I, BisIndolmaleimide IX, Go6983, but not by Go6976 in MDA MB 435 cells (Fig. 1j). In contrast, HT1080 cells treated with erastin can also be rescued by BisIndolmaleimide I, BisIndolmaleimide IX, and Go6983, yet RSL3 induced ferroptosis can only be rescued with BisIndolmaleimide I and Go6983 (Supplementary Fig. 3c). As we systematically assessed the specificity of each inhibitor on PKC isoforms, our results indicated both PKCβII and PKC-γ are involved with ferroptosis depending on different cell lines and ferroptosis inducers which confirmed previous studies.^24^

### Phosphatidylserine exposure and membrane perforation as potential biomarkers for ferroptosis

**Fig. 2:**
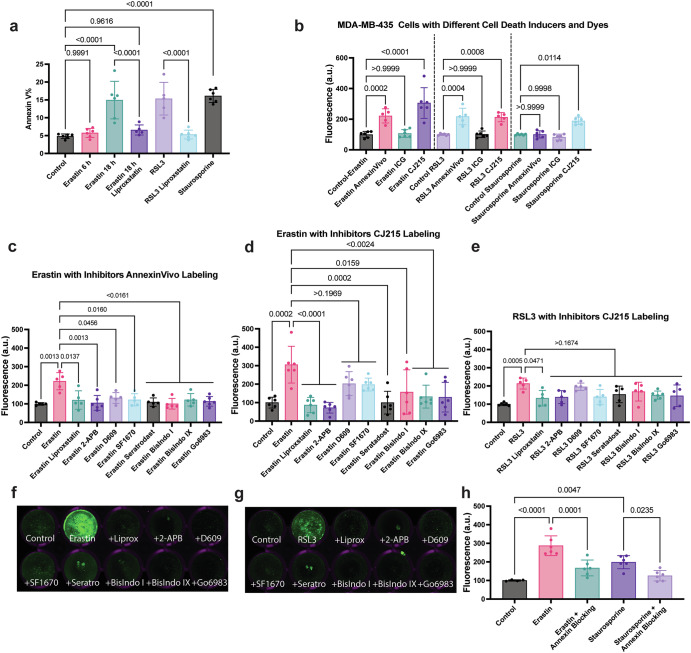
Ferroptosis induction leads to phosphatidylserine exposure and can be detected with commercially available dyes AnnexinVivo and CJ215. **a** An Annexin V staining of cells treated with erastin (15 μM) for 6 and 18 h and RSL3 (1 μM) treated for 2 h either with and without Liproxstatin (2 μM) shows an increased fluorescence upon ferroptosis induction which can be decreased with liproxstatin. 18 h staurosporine treatment is used as a positive control. **b** Measuring the fluorescence levels in MDA MB 435 cells treated with erastin, RSL3 and staurosporine using three different dyes AnnexinVivo, ICG and CJ215 showing the effectiveness of CJ215 over the other contrast agents. **c** Measurement of fluorescence upon 24 h of erastin treatment (10 μM) along with ferroptosis inhibitors using AnnexinVivo staining. **d** Measurement of fluorescence upon 24 h of erastin treatment (10 μM) along with ferroptosis inhibitors using CJ215 staining. **e** Measurement of fluorescence upon 6 h of RSL3 treatment (1 μM) along with ferroptosis inhibitors using CJ215 staining. **f** Representative image of erastin-treated cells with and without ferroptosis inhibitors using CJ215 staining. **g** Representative image of RSL3-treated cells with and without ferroptosis inhibitors using CJ215 staining. **h** Fluorescence intensity in CJ215-stained cells treated with erastin and staurosporine with or without addition of Annexin V proteins show decreased fluorescence upon Annexin V blocking. *N* = 5 pooled from three independent experiments. Mean ± s.d. *p* values are reported above the lines (Ordinary one-way ANOVA with Tukey’s multiple comparisons).

As we found shared pathways between platelet activation and ferroptosis, such as TXA_2_ receptor activation and PIP_2_-PLC-IP_3_ receptor and PKC pathway, we asked whether phospatidylserine exposure as a hallmark of platelet activation^25^ also occurs during ferroptotic cell death. This change would not be detected with existing lipidomics approaches as they can only detect the changes in lipid content, not the localization or accessibility of lipids in the membrane. Phosphatidylserine exposure is typically monitored using Annexin V protein conjugated to an organic dye, which would bind to the outer membrane in apoptotic cells. The Annexin V protein conjugated with a dye is used in conjunction with a membrane-impermeable dye that tests membrane integrity in cells undergoing cell death. In the case of apoptosis, phosphatidylserine exposure occurs when the membrane is intact, so apoptotic cells only show an increase in Annexin V staining without an increase in the membrane impermeable dye. In contrast, ferroptosis-damaged membranes with nano-sized gaps lead to an increased uptake of membrane-impermeable dyes within the early stages of ferroptosis and continue to increase towards the late stages with phosphatidylserine exposure. In that sense, our approach resembles imaging necrosis through Annexin V staining, where Annexin V protein can enter the cells via pores. When we stained early and late-stage ferroptotic cells with Annexin V, we observed an increase in signal during late-stage erastin-induced ferroptosis, which can be inhibited with ferroptosis inhibitor liproxstatin. Similarly, 2 hours of RSL3 treatment resulted in an increase in Annexin V staining that was reduced by liproxstatin (Fig. 2a). Since this increase in Annexin V staining occurred in the late stages of ferroptosis, this change can result from either i) exposure of phosphatidylserine to the outer leaflets and ii) Annevin V-dye conjugate passing through the 5 nm sized gaps^16^ and binding to phosphatidylserine molecules on the inner leaflet. However, as these mechanisms are not mutually exclusive, both can play a role to label phosphatidylserine during the late stages of ferroptosis in a characteristic pattern different from apoptosis.

To further characterize this phosphatidylserine labeling in ferroptotic cells, we utilized a commercially available dye called AnnexinVivo. It should be noted that AnnexinVivo is Annexin A5 protein conjugated to near-infrared fluorescent dyes from PerkinElmer which also suitable for apoptosis staining in vivo according to the manufacturer. In contrast, Annexin V stain is Annexin A5 protein conjugated to FITC. Recent studies in our lab identified CJ215^26^ as a novel pan-cancer agent that is chemically derived from indocyanine green (ICG) and offers a unique opportunity for cancer targeting. Our previous studies revealed that, unlike ICG which accumulates into tumors via the Enhanced Permeability and Retention (EPR) effect, CJ215 binds to serum proteins such as albumin^26^ upon in vitro incubation or in vivo injection reminiscent of other carbocyanine dyes^27,28^. These dye-protein complexes are taken up avidly by tissues with high metabolism, such as tumors or immune cells, as energy sources, leading to a high tumor-to-muscle ratio uptake—similar to the uptake of glucose in tumors. Our previous studies also demonstrated that CJ215 could bind to cellular membranes of cells undergoing apoptosis and can be used to label wounds where cell death and platelet activation occurs^26^. This led us to speculate whether CJ215 can also be used as an imaging agent for ferroptosis. To ascertain whether AnnexinVivo and CJ215 can be used to image cell death, we tested the in vitro labeling capabilities of AnnexinVivo, CJ215 and ICG (as control) of cells treated with ferroptosis inducers erastin, RSL3 and apoptosis inducer staurosporine. We treated cells with erastin or staurosporine for 24 h or with RSL3 for 6 h (to induce late-stage ferroptosis) and added the dyes AnnexinVivo, CJ215 or ICG in the last 6 h of this incubation period. Following incubation and washing, cells were imaged with the Licor Odyssey system for quantification. ICG levels did not increase in any of the cells treated with either ferroptosis or apoptosis inducers, indicating the loss of membrane structural integrity during ferroptosis does not lead to increased non-specific dye uptake unless dye molecules have a target they can bind to in order to avoid being washed out and remain within the cells during the wash step. In contrast, both AnnexinVivo and CJ215 showed increased uptake with RSL3 and erastin treatment and can be used for monitoring ferroptosis in vitro, while, surprisingly, CJ215 was the only dye that increased signal upon staurosporine treatment and can also be used as an apoptosis imaging agent (Fig. 2b). These results are also seen in HT1080 cell lines treated with the same dyes and cell death inducers (Supplementary Fig. 4). It is also important to note that since ferroptosis causes gaps in lipid membranes as well as phosphatidylserine exposure, there are more targets for AnnexinVivo and CJ215 to bind, making them more effective labels for ferroptosis over apoptosis (which is also evident in their moderate labeling ability in staurosporine-treated cells. Overall, our results indicate CJ215 can serve as an optical imaging agent for monitoring both ferroptosis and apoptosis, being superior to AnnexinVivo.

Next, we tested whether ferroptosis inhibition can be monitored using CJ215 and AnnexinVivo dyes. Erastin treatment of MDA-MB-435 cells led to increased AnnexinVivo binding, which can be inhibited with several novel ferroptosis inhibitors identified in this study, as well as those previously known^1^ (Fig. 2c). In contrast, AnnexinVivo did not show an increase in HT1080 cells treated with erastin (Supplementary Fig. 5a), but did show an increase in HT1080 cells treated with RSL3 (Supplementary Fig. 5b). These results suggest that AnnexinVivo can serve as an important ferroptosis labeling agent, but it may show different characteristics depending on the cell type such as MDA-MB-435 and HT-1080 cell lines we used in this study. Next, we tested whether erastin treatment increases CJ215 binding and observed an increase in CJ215 fluorescence upon ferroptosis induction (Fig. 2d, f), which can be prevented with ferroptosis inhibitors. Similarly, RSL3-induced ferroptosis also led to an increase in CJ215 accumulation in the cells, which was reduced by ferroptosis inhibitors (Fig. 2e, g). CJ215 staining also functioned in HT1080 cells with both ferroptosis inducers erastin and RSL3 (Supplementary Fig. 6a, b), indicating the flexibility of this dye over current commercial solutions. In order to prove that CJ215 also binds to phosphatidylserine species, we performed a blocking assay where we compared CJ215 staining of erastin-induced ferroptosis and staurosporine-induced apoptosis in the presence or absence of Annexin V protein (distinct from Annexin V staining) to block phosphatidylserine binding. CJ215 labeling of the erastin and staurosporine-treated cells was clearly decreased upon prior Annexin V incubation, indicating phosphatidylserine binding is one of the mechanisms contributing to CJ215 retention (Fig. 2h).

Overall, our previous publications on lipid membrane binding of CJ215 in cells undergoing apoptosis^26^ along with our blocking studies, indicate that CJ215 binds to phosphatidylserine. While apoptosis and ferroptosis share phosphatidylserine exposure, it is important to make the distinction that while this labeling occurs in apoptosis only on the outer membrane leaflet through exposure (flipping) of phosphatidylserine exposure on the outer leaflet, in ferroptosis, small molecule dyes such as CJ215 can also enter into the cells through nano-sized gaps in the membrane induced by ferroptosis and also bind to phosphatidylserine in the inner leaflet of the membrane or on mitochondria and endoplasmic reticulum. Yet, ICG did not lead to an increased accumulation in cells treated with ferroptosis inducers, which discards the possibility that increased CJ215 staining is only due to nonspecific uptake of a dye across damaged membranes. The most likely mechanism of CJ215 labeling in ferroptotic cells is therefore utilization of gaps on the membrane to bind phosphatidylserine molecules (or, of course, additional unknown targets) on the outer and inner leaflets. Given that CJ215 can target both of the leaflets, this could also explain the reason why CJ215 performs better with ferroptosis inducers compared with apoptosis inducer staurosporine. Collectively, these results show that CJ215 uses unique mechanisms in apoptosis and ferroptosis to bind to cellular structures and is retained on the cell membrane, which can be utilized to image the effect of apoptosis or ferroptosis inducers and their inhibitors.

### In vivo imaging of ferroptosis-based therapies

**Fig. 3:**
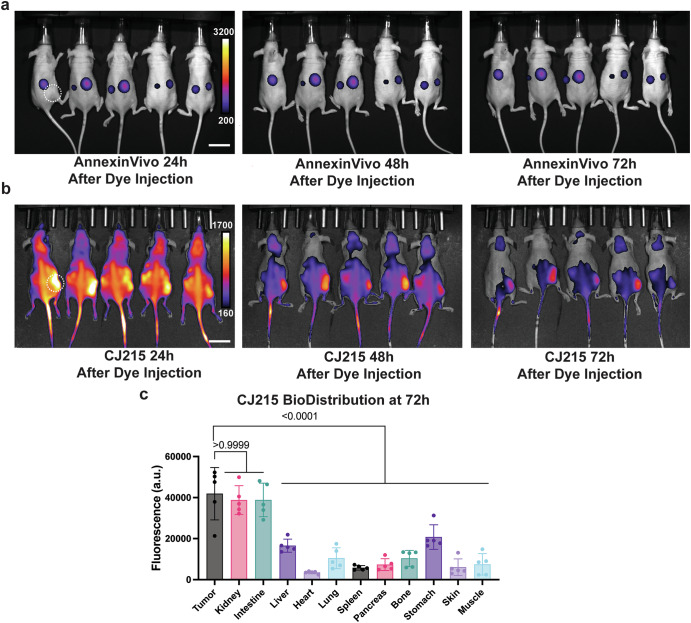
CJ215 offers enhanced targeting and contrast in vivo for tumor labeling. **a** IVIS imaging of HT1080 xenograft tumor bearing nude mice injected with AnnexinVivo dye shows kidney localization at 24, 48 and 72 h, **b** IVIS imaging of HT1080 xenograft tumor bearing nude mice injected with CJ215 dye shows tumor localization at 24, 48 and 72 h with decreasing background over time. **c** Biodistribution of CJ215 at different organs of HT1080 xenograft tumor-bearing nude mice at 72 h shows increased fluorescence in tumor, intestine and kidney, showing the excretion routes of CJ215, *n* = 5 mice. Intestinal signal is also in part attributable to chlorophyl fluorochromes in the animals’ food. Mean ± s.d. *p* values are reported above the lines (Ordinary one-way ANOVA with Tukey’s multiple comparisons). Scalebar 50 mm.

At the moment, there are no optical imaging agents that can monitor ferroptosis therapy, while glutamate-based radiotracers have been applied for monitoring tumor redox status^29^. This unmet need led us to test both AnnexinVivo and CJ215 in vivo to assess their tumor labeling ability and their capacity to monitor ferroptosis treatment response in mice upon therapy with ferroptosis inducers. To understand the general tumor-targeting capabilities of these imaging agents, we generated subcutaneous xenograft HT1080 models in the flank region of the mice, which were then injected with either AnnexinVivo or CJ215 without inducing ferroptosis to determine dye biodistribution. Our results showed that 24 h post-injection Annexin Vivo demonstrated non-specific uptake in kidneys and other organs and was retained in these areas throughout the 3-day imaging period. Crucially, AnnexinVivo showed no tumor uptake (Fig. 3a, Supplementary Fig. 7a-c). In contrast, as shown previously, CJ215^26^ readily accumulated in tumor regions within 24 h, followed by gradual clearance from other organs while being retained in the tumor site (Fig. 3b, Supplementary Fig. 7d-f). It should be noted that the intestine signal most probably comes from the food, whereas the kidney signal comes from the CJ215 signal. These results collectively show the power of CJ215 over AnnexinVivo in tumor labeling.

**Fig. 4:**
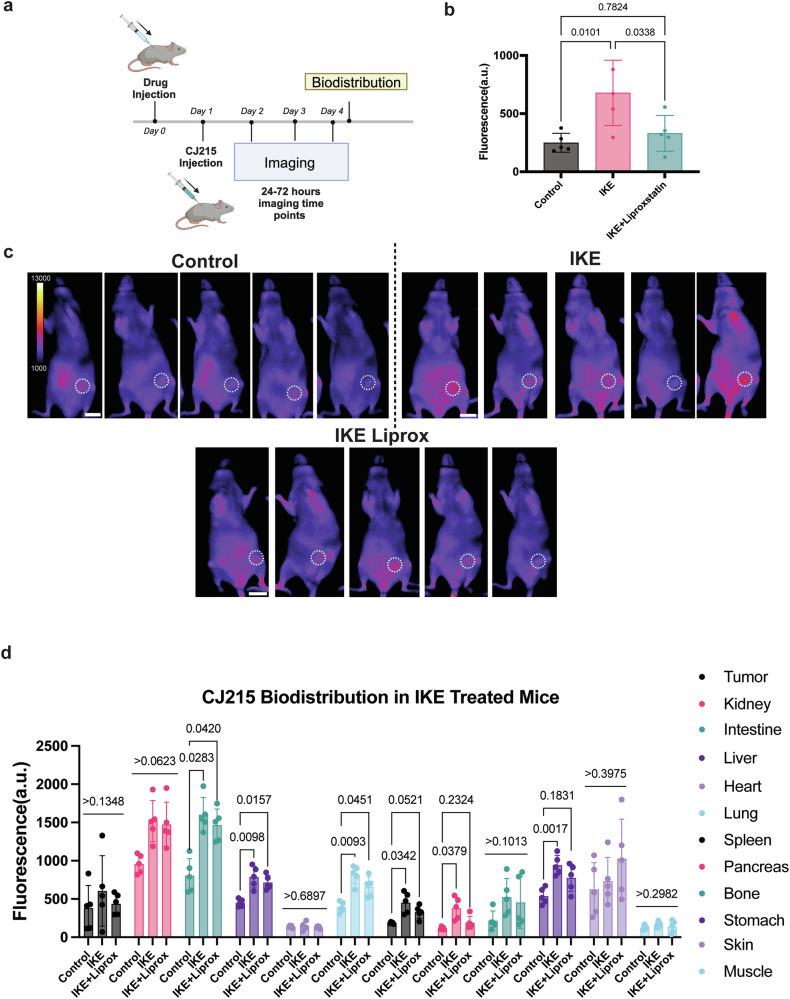
In vivo CJ215 can detect ferroptosis in vivo for monitoring ferroptosis therapies or side effects of ferroptosis drugs. **a** Scheme outlining the experimental workflow for ferroptosis imaging from the IKE injection to imaging and biodistribution studies done in Biorender. **b** Quantification of tumor uptake at 24 h after dye injection by subtracting the background CJ215 accumulation in each mouse across control, IKE treated and IKE+Liproxstatin treated groups. *n* = 5 mice. **c** Comparison of Contrast-to-Noise Ratio of CJ215 imaging in individual tumor-bearing mice treated with vehicle (control group), IKE and IKE+Liproxstatin imaged 24 h after dye injection. **d** Quantification of CJ215 fluorescence in different organs at 72 h after dye injection, indicating ferroptosis levels in mice groups treated with vehicle(control), IKE and IKE+Liproxstatin, indicating off-target effects of IKE treatment in inducing ferroptosis in specific organs, Mean ± s.d. *p*-values are reported over the lines (Ordinary one-way ANOVA with Tukey’s multiple comparisons). Scalebar 20 mm.

While CJ215 had increased uptake in tumor sites compared to AnnexinVivo, another consideration was the rate of tumor uptake and clearance. Our results showed HT1080 xenograft tumors had a lower Contrast-to-Noise Ratio (CNR) that reached an appreciable level only around 72 h after dye injection (Fig. 3c, Supplementary Fig. 8). Given ferroptosis typically occurs within the 24–48 h range after erastin addition, and dye accumulates in cells already undergoing ferroptosis, this slow accumulation and late CNR increase in HT1080 tumors prevented analysis in the time of interest. To properly image tumor responses with increased CNR at the correct imaging window, we wanted to employ an alternative xenograft orthotropic model that achieves a high CNR faster, which led us to utilize MDA-MB-435 cell lines injected into mammary fat pads. In this study, we utilized Imidazole Ketone Erastin (IKE)^30^ which was derived from erastin as a ferroptosis inducer that has better metabolic stability and potency, optimized for in vivo use. We injected IKE either alone, or in combination with ferroptosis inhibitor liproxstatin, and waited 24 h for the drug to reach the tumor site and induce ferroptosis. After this initial 24 h, we injected CJ215 to the IKE treated, IKE+Liproxstatin treated and the control groups, and imaged the mice in 24 h intervals until 72 h post dye injection (Fig. 4a). At the end of the imaging experiment at 72 h post dye injection, we sacrificed the mice to study dye uptake and clearance routes.

Our results revealed, that injection of IKE or rescue with liproxstatin does not increase tumor uptake in the case of AnnexinVivo imaging with HT1080 tumors as the dye predominantly goes to kidneys (Supplementary Fig. 9). In contrast, we observed an increased retention of CJ215 in tumors of IKE treated mice compared with control mice within 24 h of dye injection, which can be inhibited by addition of ferroptosis inhibitor liproxstatin (Fig. 4b, c). This difference disappears at imaging time points 48 and 72 h post dye injection where cancer cells die and accumulated CJ215 is cleared from the tumor site (Supplementary Fig. 10a-c). When we performed the biodistribution assays at 72 h post-dye injection, we did not see any difference in CJ215 levels in tumors as cells undergoing ferroptosis already died. In contrast, we observed significant increases of CJ215 accumulation in IKE and IKE+Liproxstatin treated mice in specific organs such as intestine, liver and lung (Fig. 4d and Supplementary Figs. 11 and 12).

**Fig. 5:**
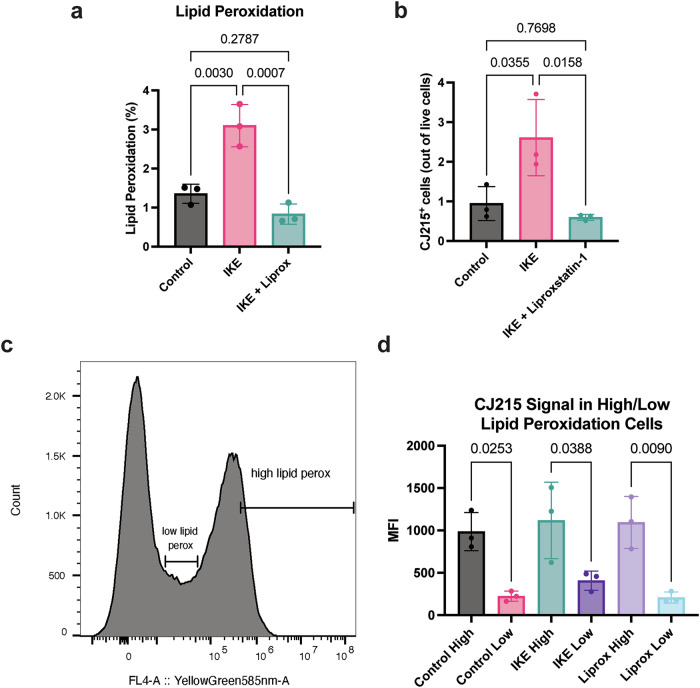
Ex vivo analysis comparing lipid peroxidation and CJ215 uptake in mouse kidney. **a** Quantification of lipid peroxidation levels in the live cell populations of kidney cells from mice groups serving as Control, IKE treated, IKE+Liproxstatin treated. **b** Quantification of CJ215 positive cells from the live cell populations of the same group. **c** Bodipy C11 signal indicating lipid peroxidation and their histogram distribution where we selected low and high lipid peroxidation populations marking the 10% of cells on either end. **d** Comparison of mean CJ215 fluorescence signal in low and high lipid peroxidation populations in each mouse group, indicating an increased CJ215 uptake in cells with higher lipid peroxidation. *N* = 3 mice per group.

We also wanted to confirm whether increased CJ215 uptake is correlated with increased lipid peroxidation in mice. To achieve this, we used control, IKE, IKE+Liproxstatin treatments, and injected CJ215 to mice. Later, we isolated and dissociated the kidney and stained the cells with Bodipy C11. Our results showed increased lipid peroxidation in IKE treated group which can be prevented with Liproxstatin (Fig. 5a). Later, we compared the CJ215 uptake between mice groups in live cells and observed CJ215 positive cells were significantly higher in IKE group compared with the other two (Fig. 5b). Finally for a direct comparison, we designated “low lipid peroxidation population” at the lower 10% of the second peak in the Bodipy C11 distribution (Supplementary Fig. 13 for our gating strategy) whereas we designated higher 10% of the second peak as “high lipid peroxidation population”(Fig. 5c). When we compared the mean fluorescence intensity of CJ215 signal for each group, we saw all groups, even tissues not treated with IKE showed an increased accumulation of CJ215 in high lipid peroxidation populations (Fig. 5d). These results provide an ex vivo confirmation where CJ215 can label cells with increased lipid peroxidation even at the basal lipid peroxidation levels.

These results indicate that while IKE is a potential anti-tumor agent, it can also lead to off-target effects and induce ferroptosis in other organ types which rely on System Xc^−^ as a part of their cellular homeostasis. While liproxstatin co-injection significantly decreased most of these organ accumulations (with the exception of intestines, where some signal might be derived from chlorophylls in the diet), it was not sufficient to protect the entire mouse. Overall, these results indicate, beyond tumor imaging, CJ215 accumulation also offers unique opportunities to test ferroptosis in physiological settings and can be employed to study side effects of ferroptosis inducing drugs or effectiveness of ferroptosis inhibitors.

## Discussion

In this study, we explored how ferroptotic cell death is accompanied by changes in membrane dynamics and how lipid peroxidation accumulation results in membrane damage, which we characterized with different sizes of quantum dots and various dyes, taking advantage of their size-dependent emission spectra changes and binding characteristics. We show that membrane damage opens nano-sized gaps using machinery similar to platelet activation. Many platelet activation inhibitors in PIP_2_-PLC-IP_3_-PKC pathways can also be utilized as ferroptosis inhibitors. It is interesting to note how platelet activation is also implicated in stroke and plaque formation, which are shown to have underlying ferroptotic components^31^. These similarities between platelet activation and ferroptosis can also explain why ferroptosis is not directly an immunogenic cell death despite a loss of membrane integrity, unlike necroptosis^32^. We have further shown how ferroptosis-induced nano-sized cell membrane gaps during late-stage ferroptosis can lead to Annexin V binding either through phosphatidylserine exposure or through traversing cell membrane and binding to phosphatidylserine molecules on the inner leaflet. We reasoned phosphatidylserine targeting approaches, such as Annexin V-based assays would serve as potential ferroptosis labeling agents, and we demonstrated this using both conventional Annexin V stain as well as a commercially available in vivo imaging agent called AnnexinVivo. Despite their use in the in vitro setting, annexin-based imaging agents were not highly successful in detecting apoptotic cells in vivo in the past^33^, and our experience with AnnexinVivo revealed non-specific uptake by other organs and limited tumor labeling. Therefore, we turned to a novel carbocyanine dye, CJ215, that binds to serum proteins both in vitro and in vivo to act as a transient passenger for tumor uptake. Given the excess nutrient demands of tumors compared with the rest of the body^34^ these dye-protein complexes preferentially accumulate in the tumor sites. In the ferroptotic and apoptotic cancer cells, however, CJ215 shows even a more pronounced accumulation which can be prevented using unlabeled Annexin V protein as a blocking agent. It is important to note that these two mechanisms are not mutually exclusive and can work in tandem where dye-albumin complexes are essential for bringing the CJ215 into the tumor site and phosphatidylserine binding can lead to increased retention on cancer cells. Overall result would be high CNR in tumors undergoing cell death which can be utilized for therapy monitoring.

In vivo application of CJ215 still suffers from several challenges, given that it only binds to the cells during the late stage of ferroptosis. This requires a comprehensive knowledge of the biodistribution of ferroptosis inducers, the response time of tumors to the ferroptosis inducers, as well as dye uptake and clearance, all of which would contribute to the success of CJ215 as a therapy monitoring dye. While our efforts were successful in image changes in tumors undergoing ferroptosis in MDA-MB-435 tumors, HT1080 xenografts had slow clearance which increased the background and lowered tumor-selective Contrast-to-Noise Ratios. Thus, future applications of CJ215 would require an in-depth knowledge of tumor type, ferroptosis inducer and imaging agent, and their successful coordination for an optimized protocol that is unique for each situation. Finally, we are aware phosphatidylserine exposure and membrane defects are not unique biomarkers for ferroptosis, and we do not imply CJ215 imaging can be used to prove a drug is in a fact inducing only ferroptosis. Yet, we have established a ferroptosis imaging approach that is applicable to an in vivo setting which was greatly needed. Here, a known ferroptosis inducer, combined with a suitable rescue experiment via ferroptosis inhibitors such as liproxstatin, can offer unique insights into in vivo ferroptosis biology. We believe CJ215-based imaging approaches have wide-ranging applications not only for tumor labeling, but also for reporting side effects of ferroptosis inducers in diverse organ systems along with a cancer therapy monitoring approach for both apoptosis and ferroptosis-based treatment strategies.

## Methods

### Cell culture

MDA-MB-435 and HT1080 cells were obtained from American Type Culture Collection(ATCC). MDA-MB-435 cell line was cultured in RPMI 1640 Medium(Thermo Fisher), 10% Fetal Bovine Serum (FBS) and 1% Penicilin-Streptomycin (Pen-Strep) Solution. HT1080 cell line was cultured with DMEM medium (Thermo Fisher) with 10% FBS and 1% Pen-Strep. Media was changed in every 3 days to ensure nutrient availability and cells were passaged at 80% confluence with 0.05% Trypsin EDTA (Thermo Fisher). Cell viability and health were constantly monitored during the course of the study.

### Quantum dot uptake

Cultured cells were washed with Phosphate Buffer Saline(PBS) and trypsinized. Cells were centrifuged to remove excess trypsin and were resuspended in their respective culture media. Cells were placed in 24-well plates with 75,000 cells per well with a media volume of 500 μL. The cells were incubated at 37 °C with 5% CO_2_ levels overnight for surface attachment. Each well was treated with either vehicle or erastin(10 μM) (Cayman Chemicals) for 6 or 18 h. After the incubation, the cells were washed with PBS and trypsinized for flow cytometry preparation. They were subsequently centrifuged for 5 min at 1000 rpm and resuspended in PBS containing 2% FBS. Cells were centrifuged again and resuspended again in PBS-2%FBS. Then, the cells were incubated with DAPI (1 μM), carboxyl-coated QD450, QD550, QD650 quantum dots (SigmaAldrich) (1 μL from the stock solution) separately for 5 min on ice and were analyzed in flow cytometry (MACS Quant). Given the distinct spectral nature of quantum dots and dyes, we could distinguish each quantum dot type in different channels. We followed the 5 min incubation protocol on ice to prevent non-specific uptake and employed same surface-coated Quantum dot types to ensure only size remains a factor in quantum dot uptake. We used the negatively charged carboxyl-coated quantum dots to prevent non-specific charge-based interactions with the cell membrane. We also normalized the QD uptake to the vehicle-treated cells to ensure nonspecific cell surface binding is accounted for. The change in cellular fluorescence was analyzed using FlowJo program. Quantum dot sizes were also confirmed using Malvern PanAnalytical Zeta Sizer measurements.

### Cytotoxicity

Cells were trypsinized and seeded on 96-well plate with 10,000 cells per plate in 150 μL. The cells were incubated at 37 °C with 5% CO_2_ levels overnight for surface attachment. Only the center 60 wells were used for the assays to prevent the edge effects. The cells were treated with erastin(10 μM) or RSL3(2 μM)(Cayman Chemicals) in the presence and absence of the inhibitors and activators. 2-APB, D609, SF1670, and Rotenone were also purchased from Cayman Chemicals and utilized with final concentrations of 2 μM, 5 μM, 0.2 μM and 1 μM, respectively. The concentrations of the other chemicals are given in Supplementary Tables all of which were purchased from MedChem. Cells were treated with ferroptosis inducers and inhibitors for 24 h and cytotoxicity was measured using Cell Titer Glo2 Assay (Promega) based on the manufacturer’s instructions. The cells were incubated with Cell Titer Glo2 reagent for 10 min and this mixture was transferred to a black-walled plate to prevent cross-well light detection. The bioluminescence was measured using Molecular Devices SpectraMax iD5 Multi-Mode Microplate Reader with 1000 ms integration time with open spectral detection.

### Mitochondrial membrane potential

Cells were trypsinized and seeded on 96-well plate with 20,000 cells per plate in 150 μL. The cells were incubated at 37 °C with 5% CO_2_ levels overnight for surface attachment. Only the center 60 wells were used for the assays to prevent the edge effects. The cells were treated with erastin(10 μM) in the presence and absence of the inhibitors for 6 h. The cells were then incubated with JC10(4 μM) for 30 min and washed with PBS twice. The increase in fluorescence was measured using Molecular Devices SpectraMax iD5 Multi-Mode Microplate Reader with 560 nm excitation and 600 nm emission.

### Lipid peroxidation

Trypsinized cells were placed in 24-well plates with 75,000 cells per well with a volume of 500 μL. The cells were incubated at 37 °C with 5% CO_2_ levels overnight for surface attachment. Each well was treated with erastin(20 μM) for 6 h either with or without the ferroptosis inhibitors. For the last 30 min of the incubation, C11-BODIPY^581/591^ (Molecular Probes) (2 μM) dye was added to cell culture media based on manufacturer’s instructions. After the 30 min incubation with the dye, the cells were washed with PBS and trypsinized for flow cytometry preparation. They were subsequently centrifuged for 5 min at 1000 rpm and resuspended in PBS containing 2% FBS. Later, they were centrifuged again and resuspended in PBS-2%FBS containing DAPI (1 μM) for 5 min and they were analyzed using MACS Quant flow cytometry. C11 Bodipy dye works by increasing its green fluorescence in the presence of lipid peroxidation and we used vehicle-treated wells as a baseline to measure the relative increase in lipid peroxidation levels. The change in cellular fluorescence was analyzed using FlowJo program.

### Annexin V staining

Trypsinized cells were placed in 24-well plates with 75,000 cells per well with a volume of 500 μL. The cells were incubated at 37 °C with 5% CO_2_ levels overnight for surface attachment. The cells were treated with erastin(10 μM) for 6 or 18 h or RSL3(2 μM)(Cayman Chemicals) for 2 h in the presence and absence of Liproxstatin (1 μM). After the incubation, the cells were washed with PBS and trypsinized for flow cytometry preparation. They were subsequently centrifuged for 5 min at 1000 rpm and resuspended in PBS containing 2% FBS. Cells were centrifuged again and resuspended again in PBS-2%FBS. Then the cells were incubated for 20 min with Annexin V-FITC stain with PI dye to measure membrane integrity (MedChem) based on manufacturer’s instuctions. The cells were analyzed using MACS Quant flow cytometry. Annexin V-FITC binding indicates either phosphatidyl serine exposure or influx of Annexin V into the cell via nano-sized gaps on the cell membrane surface. The change in cellular fluorescence was analyzed using FlowJo program.

### Annevin vivo and CJ215 imaging in vitro

Trypsinized cells were placed in 24-well plates with 75,000 cells per well with a volume of 500 μL. The cells were incubated at 37 °C with 5% CO_2_ levels overnight for surface attachment. The cells were treated with staurosporine(2 μM), erastin(10 μM) for 18 h or RSL3(1 μM) for 6 h in the presence and absence of ferroptosis inhibitors. CJ215 (0.645 mM), Annexin Vivo (1:1000 dilution from stock solution) and ICG(0.645 mM), dyes were added to the cell culture media, incubated for 6 h and washed with PBS. For Annexin V blocking experiments cells were co-incubated with CJ215 and unlabeled Annexin V proteins(Abcam)(2 μg/well). The cells were imaged using Licor Odyssey CLX imager in both 680 and 800 nm channels and images were quantified using ImageJ.

### AnnexinVivo and CJ215 imaging in vivo

All mouse handling, experimentation, imaging, and housing were performed according to NIH guidelines and via approved IACUC protocols at MSKCC. Tumor xenografts were developed using 5.0 × 10^6^ HT1080 cells resuspended in 100 µL Matrigel-media mixture (1:1 ratio) and subcutaneously injected into the flank of female FoxN1^nu^ mice (6–8 weeks). Tumor growth was monitored visually, and dye injection was performed when tumors exceeded 50 mm^3^. Mice received a 2 mg/kg intravenous injection of CJ215 suspended in clinical-grade dextrose or 100 µL AnnexinVivo solution per mice as described by manufacturer’s instructions. For the therapy imaging, mice were injected with vehicle (70% PBS, 20%PEG400, 10% ethanol), IKE alone(20 mg/kg), or IKE(20 mg/kg) with Liproxstatin(10 mg/kg) 24 h before the dye injection. After the CJ215 injection(2 mg/kg), we waited another 24 h for dye clearance to achieve an optimal clearance and high signal-to-noise ratio in the tumor region. Mice were imaged using IVIS-CT with 2 s exposure time and f# of 1 with small binning using ICG filter sets(745 excitation and 840 nm emission) every 24 h. During this time mice were under isofluorane anesthesia at an induction of 3% v/v and anesthesia was maintained at 1–2% v/v in O_2_ where mice were kept on a heating pad during the course of imaging experiments. After the imaging sessions, mice were euthanized with CO_2_ and their organs were harvested. Biodistribution of the dye was assessed by isolating the organs: tumor, liver, kidneys, hearts, lungs, spleen, pancreas, bones, stomach, intestines, skin, and muscle. We performed the same imaging conditions for organ imaging, exported the tiff files that contain the fluorescence levels and analyzed the images using ImageJ/Fiji with ROIs drawn in background regions on live mice, tumor regions and individual organs. When we processed the live mice imaging experiments, we determined the background of each mice with measuring the relative dye accumulation in their chest cavity and subtracted half of this value from the total image to achieve a consistent background for the visualization. We analyzed the results using GraphPad Prism software, where we calculated the means and standard deviations of each group and performed one-way Anova with multiple comparisons or t-tests where applicable, as detailed in each figure legend.

### Ex vivo CJ215 and bodipy C11 signal comparison

Healthy nude mice were treated i.p. with IKE (20 mg/kg) and/or Liproxstatin (10 mg/kg). 24 h later 2 mg/kg of CJ215 was injected i.v. into the mice. After 24 h, the mice were imaged on the IVIS and organs were taken out for CJ215 biodistribution evaluation. The organs with high signals (the kidneys) were taken for flow cytometry analysis. Kidneys were homogenized and filtered through a 70 μm filter, after which red blood cells were lysed. The kidneys were washed with buffer (PBS with 0.5% BSA) and then incubated with 10 μM BODIPY-C11 per sample (~1 × 10^6 cells) for 1 h at room temperature. After subsequent washing, the samples were stained with 1:1000 dilution of DAPI and read on a CytoFLEX Lx cytometer.

## Supplementary information

Supplementary Information

## Data Availability

All data supporting the findings of this work are available within the article and its Supplementary Information.
