# Supplementary material for: Phosphatidylserine exposure and plasma membrane perforation as ferroptotic signatures for in vivo imaging
Source: Npj Imaging. 2025 Oct 6;3:48. doi: 10.1038/s44303-025-00110-1 (PMC12500960; doi:10.1038/s44303-025-00110-1)
Supplement: Supplementary file 1 — Supplementary Information [file 44303_2025_110_MOESM1_ESM.pdf]

Supplementary Information:
Phosphatidylserine Exposure and Plasma Membrane Perforation as Ferroptotic Signatures for In Vivo Imaging

Authors: Ali Yasin Sonay[1#], Elana Apfelbaum[2,#], Benedict Edward Mc Larney[1], Jan Grimm[1,2,3,4,5,*]

Affiliations:

[1]Molecular Pharmacology Program, Memorial Sloan Kettering Cancer Center; New York, NY, USA
[2]Pharmacology Program, Weill Cornell Medical College; New York, NY USA
[3]Department of Radiology, Radiochemistry and Imaging Sciences Service, Memorial Sloan Kettering Cancer Center; New York, NY, USA

[4]Department of Radiology, Weill, Cornell Medical Center; New York, NY, USA
[5]Molecular Imaging Therapy Service, Memorial Sloan Kettering Cancer Center; New York, NY, USA

[#]Equal contribution first author

[*]Corresponding Author: Jan Grimm (grimmj@mskcc.org)

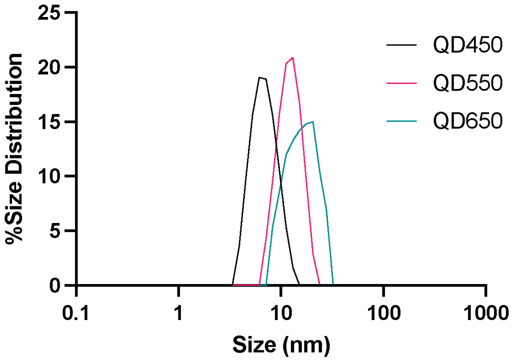

Supplementary Figure 1. Size distribution of quantum dots used in the study ZetaSizer measurements of QD450, QD550 and QD650 showing differential size distribution

a

Name of the Inhibitors and Activators	Effect of the Chemicals	Concentration
Isoprenaline	β-adrenergic receptor agonist	5 μM
Treprostinil	DP1 and EP2 agonist	5 μM
Laropiprant	DP receptor antagonist	5 μM
RO1138452	IP (prostacyclin) receptor antagonist	5 μM
Asapiprant	DP_1 receptor antagonist	5 μM
Grapiprant	EP4 receptor antagonist	5 μM
Misoprostol	Synthetic analogue of prostaglandin E1 (PGE1)	5 μM
Iloprost	Prostacyclin (PGI2) analogue	5 μM
Seratrodast	Thromboxane A2 receptor (TP) antagonist	5 μM

Table 1. List of G-Protein Coupled Receptor Modulators for Adrenergic, Prostaglandin and Thromboxane Receptors and their Derivatives

b

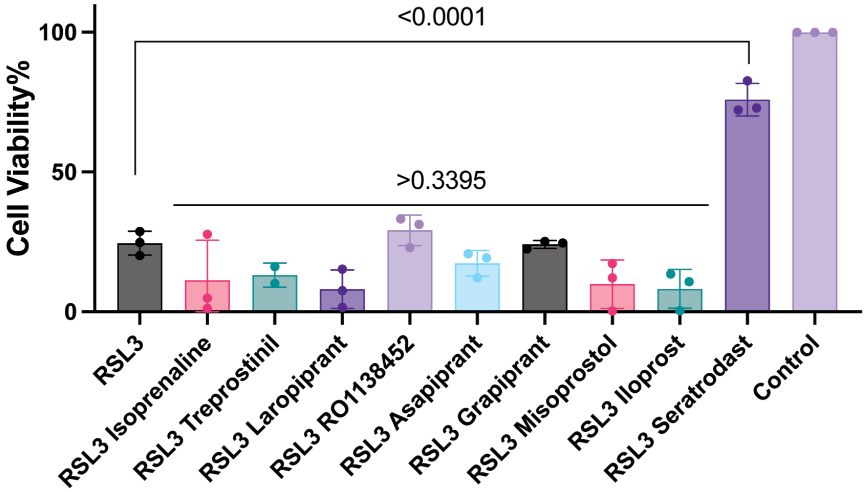

Supplementary Figure 2. Effect and concentration of different GPCR modulators tested in the study and their effect on RSL3 induced ferroptosis a) Table showing the effect and concentration of different GPCR inhibitors used in this study **b)** Cytotoxicity of RSL3 induced ferroptosis treated with Adrenergic, prostaglandin and thromboxane receptor inhibitors. For cytotoxicity assays n=3 biological replicates, Mean ± s.d. p-values are reported above the lines (Ordinary one-way ANOVA with Tukey's multiple comparisons).

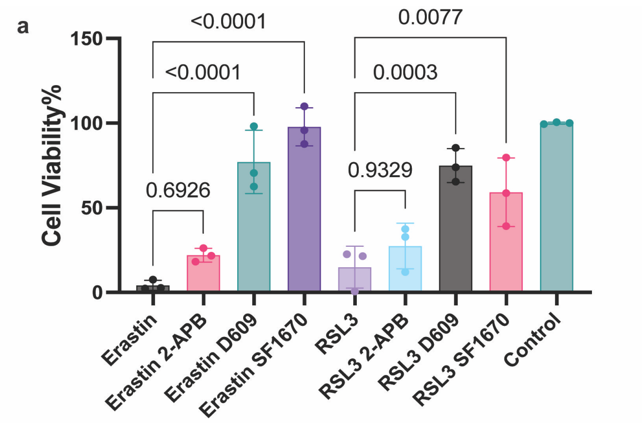

b

Name of the Inhibitors and Activators	Effect of the Chemicals	Concentration
Bisindolylmaleimide I	PKCα, PKCβI, PKCβII, and PKCγ	5 μM
Bisindolylmaleimide IX	PKC-α, PKCβI, PKCβII, PKC-γ, and PKC-ε	5 μM
Go6983	PKCα, PKCβI, PKCβII, PKCγ and PKCδ	5 μM
Go6976	PKCα, and PKCβI	5 μM

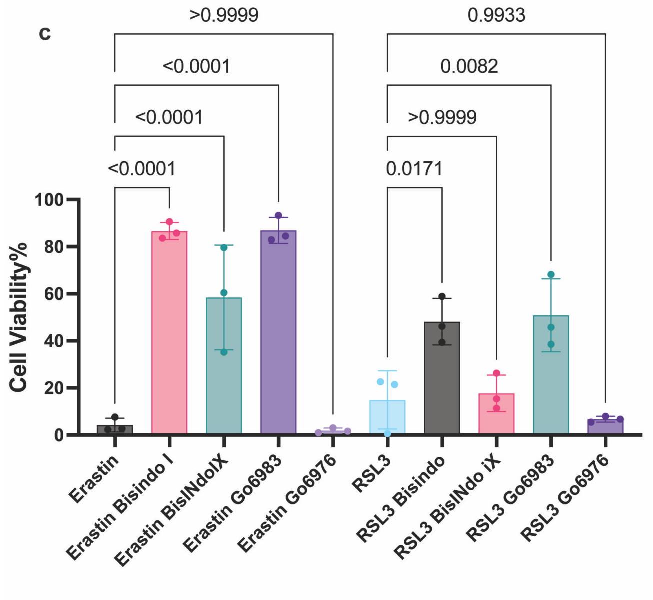

Supplementary Figure 3. Inhibitors of Platelet activation rescues ferroptotic cell death in HT1080 cell line a) IP3R, PLC and PTEN inhibitors rescue the cells from both erastin and RSL3 induced ferroptosis **b)** Effect and Concentration of different PKC inhibitors, **c)** Role of different PKC inhibitors and their effect on erastin and RSL3 induced ferroptosis. For cytotoxicity assays n=3 biological replicates, Mean ± s.d. p-values are reported above the lines (Ordinary one-way ANOVA with Tukey's multiple comparisons).

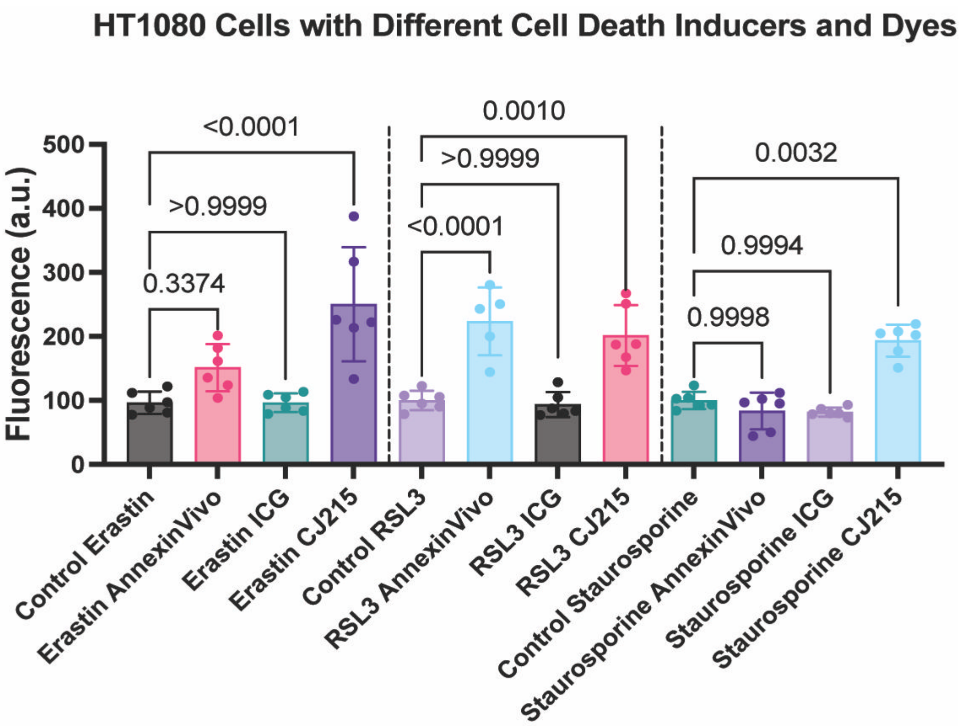

Supplementary Figure 4. Comparing the effectiveness of different dyes in HT1080 cells upon ferroptosis and apoptosis induction Measuring the fluorescence levels in HT1080 cells treated with erastin, RSL3 and staurosporine using three different dyes AnnexinVivo, ICG and CJ215 showing the effectiveness of CJ215 over the other contrast agents N=5 pooled from 3 independent experiments. Mean ± s.d. p-values are reported above the lines (Ordinary one-way ANOVA with Tukey's multiple comparisons).

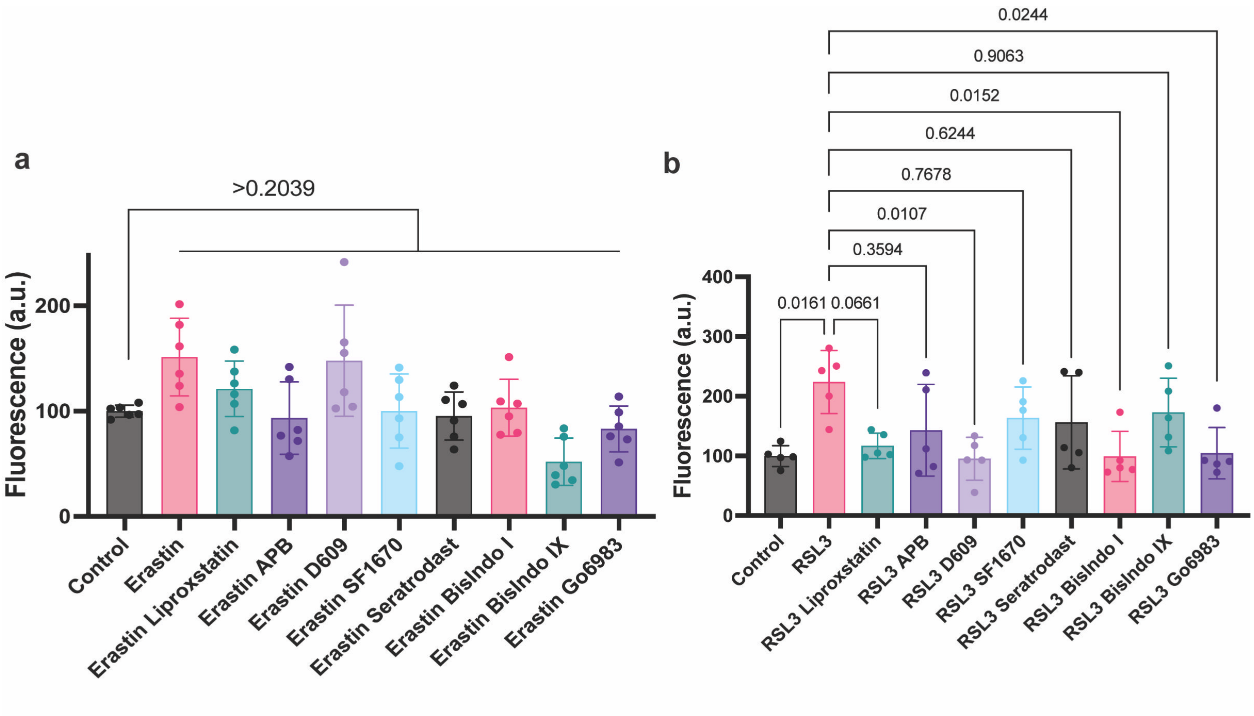

Supplementary Figure 5. Changes in Fluorescence intensity in ferroptosis inducers and inhibitors using AnnexinVivo dyes as contrast agent in HT1080 cell line a) Measurement of fluorescence upon 24 hours of erastin treatment (10 μM) along with ferroptosis inhibitors using AnnexinVivo staining **b)** Measurement of fluorescence upon 6 hours of RSL3 treatment (1 μM) along with ferroptosis inhibitors using AnnexinVivo staining N=5 pooled from 3 independent experiments. Mean ± s.d. p-values are reported above the lines (Ordinary one-way ANOVA with Tukey's multiple comparisons).

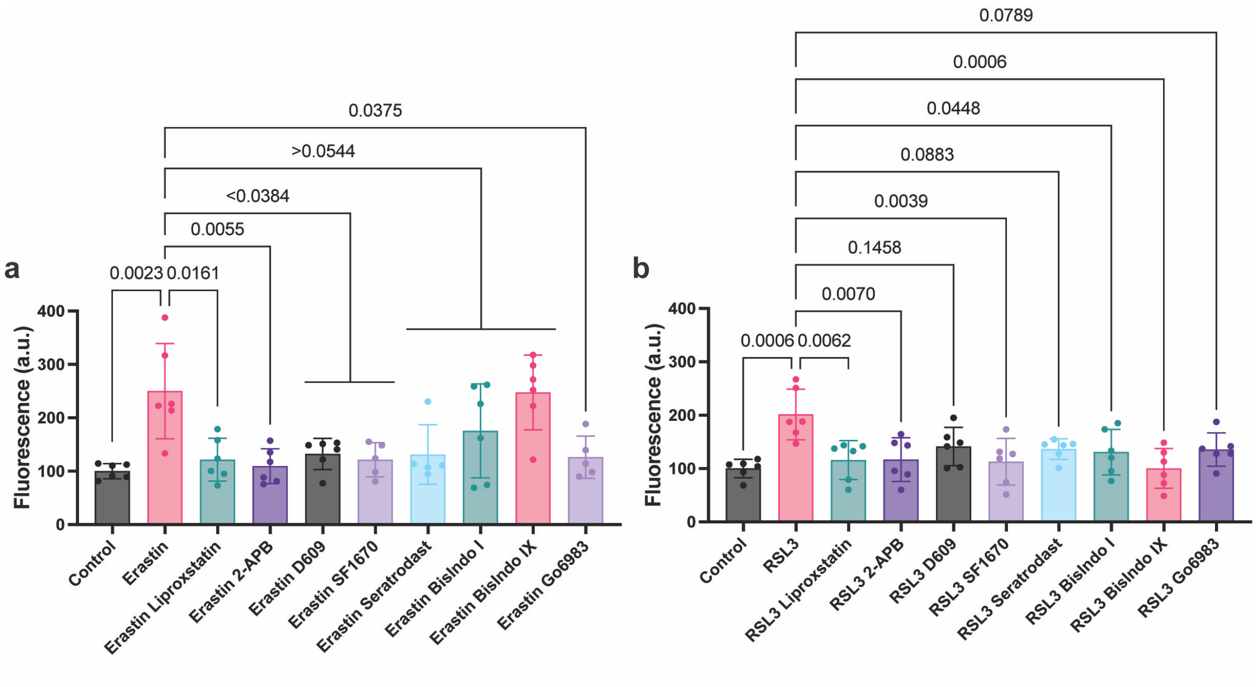

Supplementary Figure 6. Changes in Fluorescence intensity in ferroptosis inducers and inhibitors using CJ215 dyes as contrast agent in HT1080 cell line a) Measurement of fluorescence upon 24 hours of erastin treatment (10 μM) along with ferroptosis inhibitors using CJ215 staining **b)** Measurement of fluorescence upon 6 hours of RSL3 treatment (1 μM) along with ferroptosis inhibitors using CJ215 staining N=5 pooled from 3 independent experiments. Mean ± s.d. p-values are reported above the lines (Ordinary one-way ANOVA with Tukey's multiple comparisons).

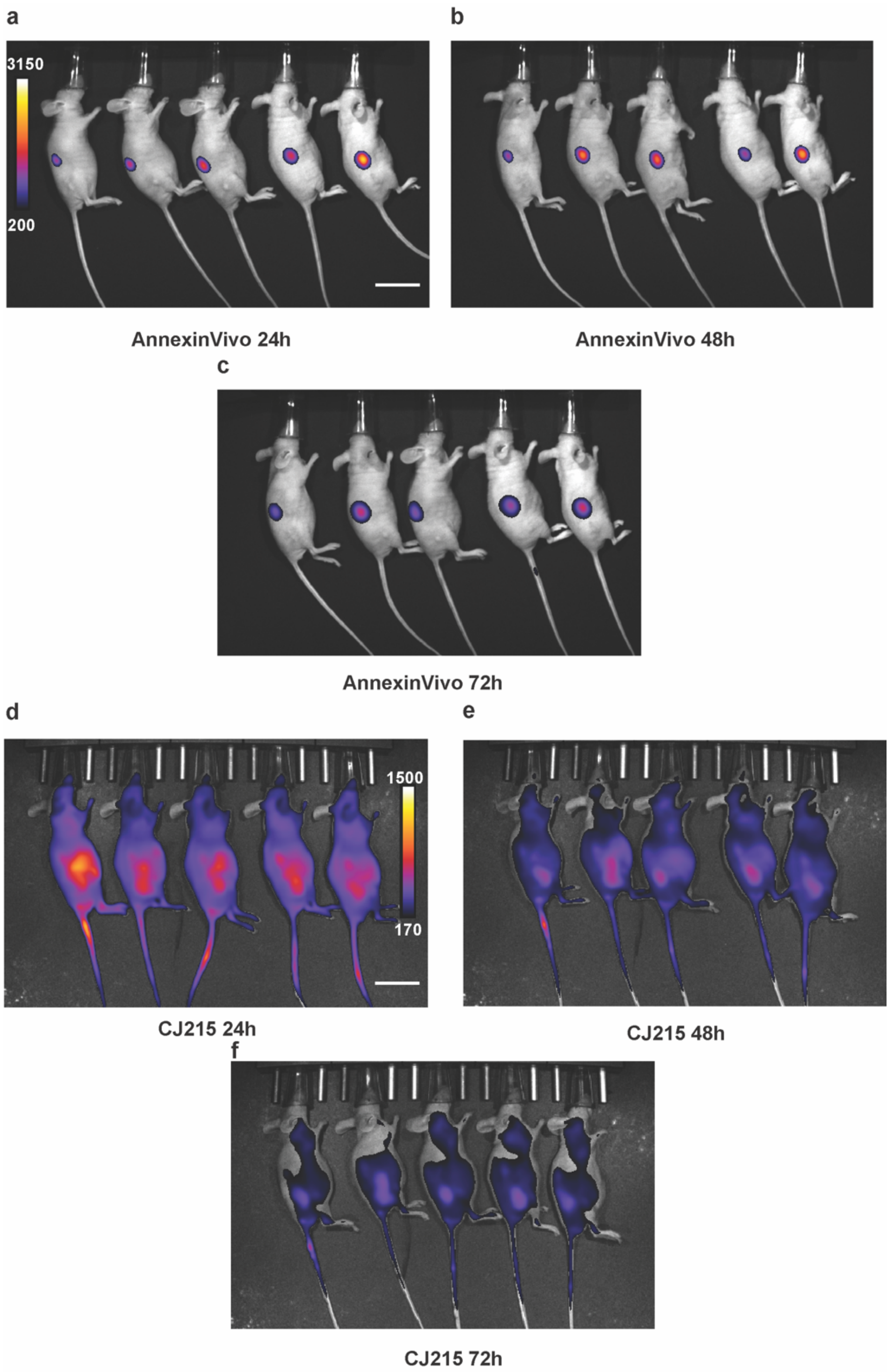

Supplementary Figure 7. Side view of AnnexinVivo and CJ215 injected mice bearing HT1080 xenograft tumors demonstrates localization of the dyes IVIS imaging of AnnexinVivo injected HT1080 xenograft tumor bearing nude mice at **a)** 24 hours **b)** 48 hours **c)** 72 hours at side view showing localization in kidney and not in the tumors, IVIS imaging of CJ215 injected HT1080 xenograft tumor bearing nude mice at **d)** 24 hours **e)** 48 hours **f)** 72 hours at side view showing localization in the tumor but with a high background in the rest of the body, n=5 mice. Scalebar 50 mm

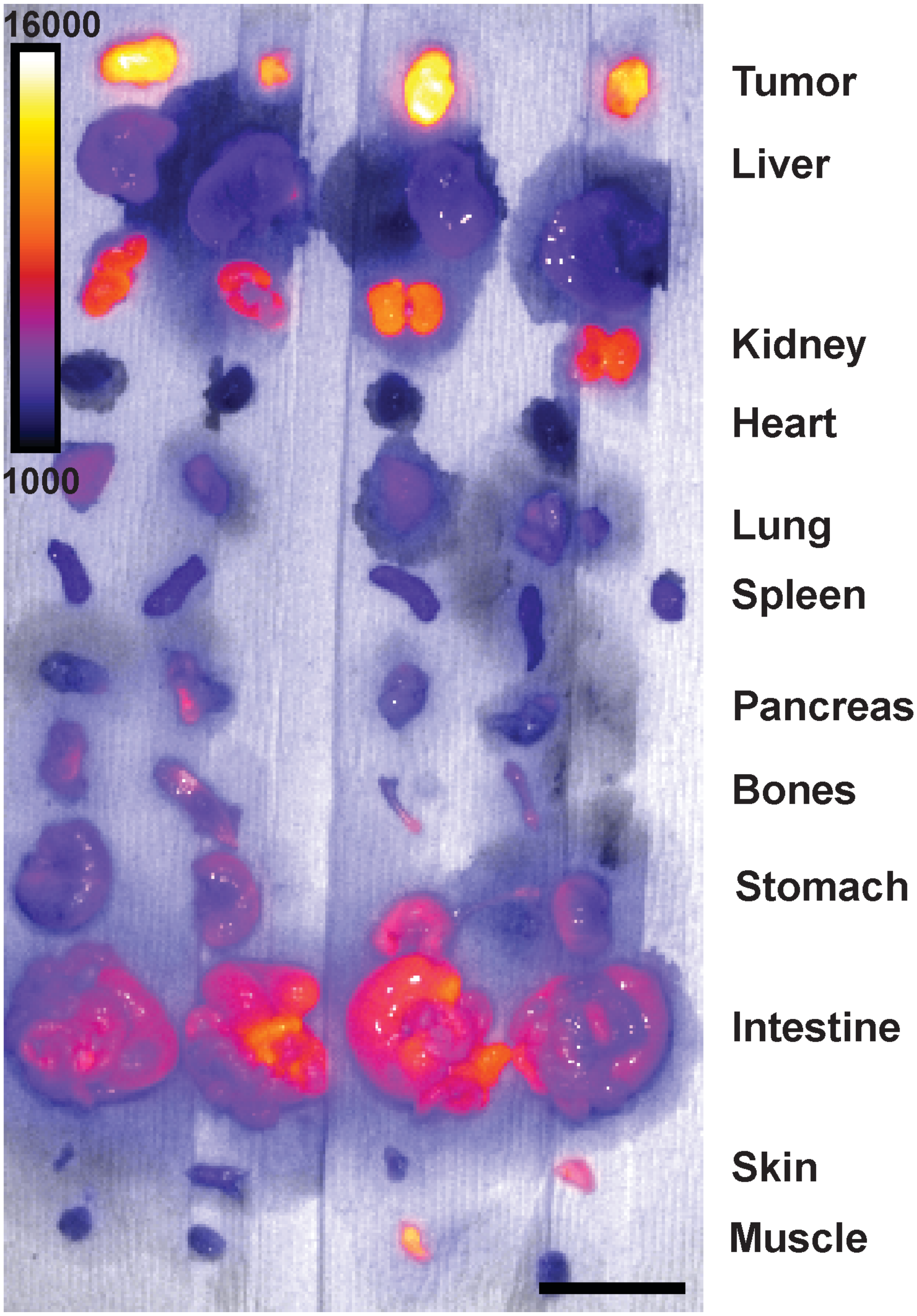

Supplementary Figure 8. Fluorescence images of the organs in CJ215 injected mice bearing HT1080 xenograft tumors demonstrating dye biodistribution Biodistribution of CJ215 in different organs in HT1080 xenograft tumor bearing nude mice 72 hours after injection shows kidneys are slowly excreting the dye over time, n=4 mice. Scalebar 50 mm.

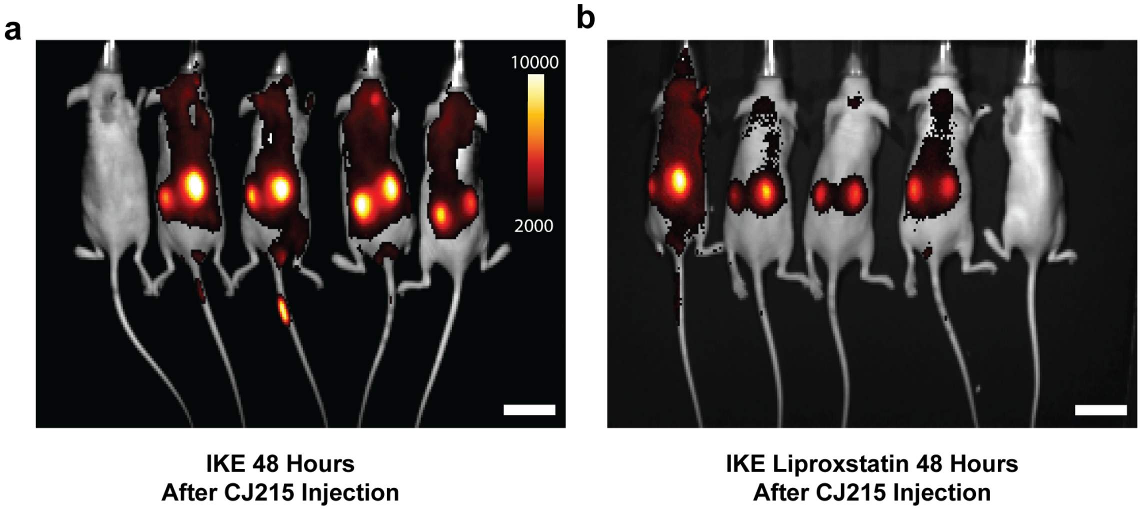

Supplementary Figure 9. AnnexinVivo Localization does not change with drug treatment. IVIS imaging of HT1080 xenograft tumor bearing nude mice injected with IKE and IKE+Liproxstatin AnnexinVivo dye shows predominantly kidney localization at 48 hours n=5 mice Scalebar 50 mm.

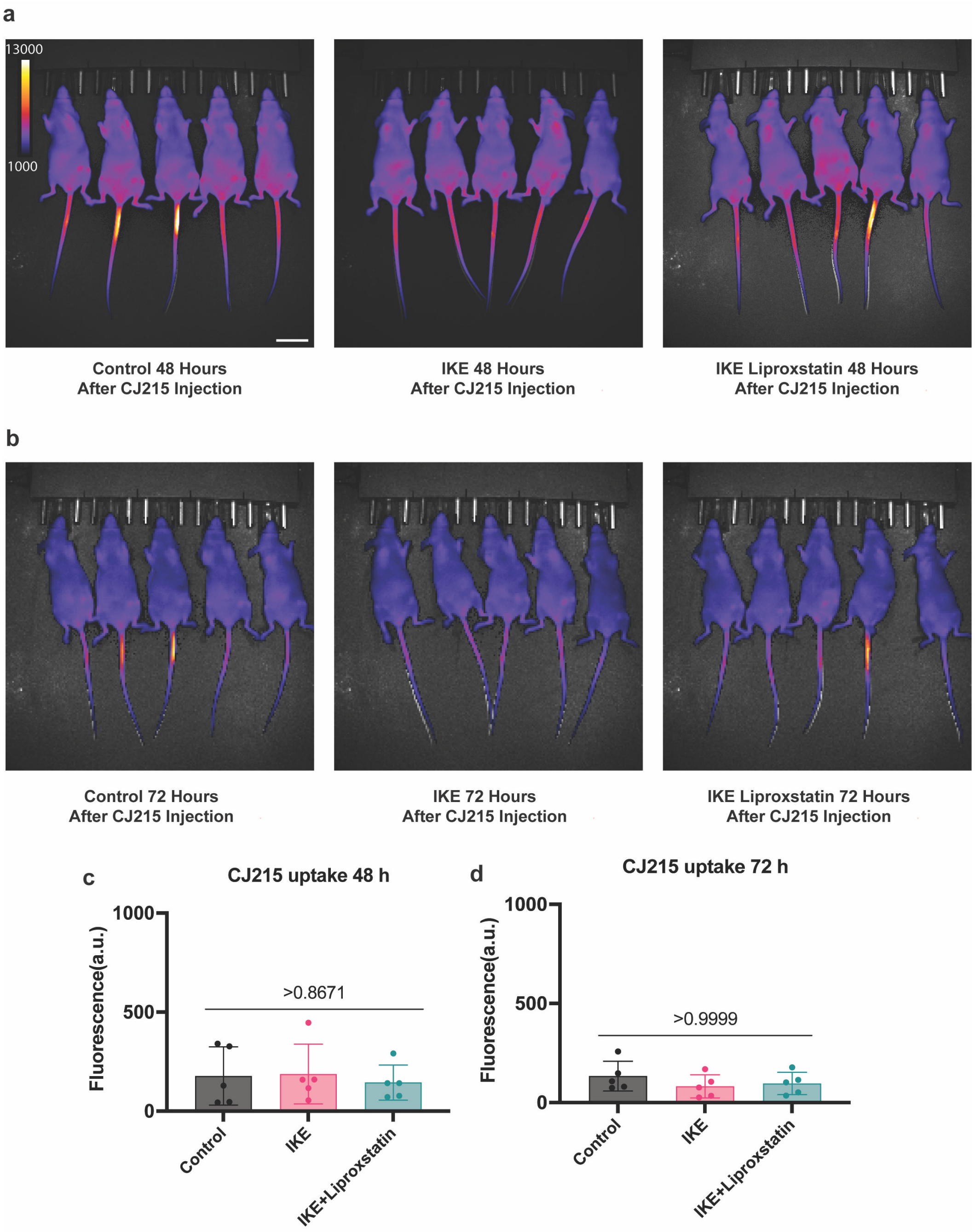

Supplementary Figure 10. Quantification of CJ215 tumor uptake at later stages of ferroptosis therapies. Imaging CJ215 tumor uptake in mice treated with vehicle(control), IKE and IKE-Liproxstatin at **a)** 48 hours post dye injection and **b)** 72 hours post dye injection**, c)** Quantification of tumor uptake between different mice groups reveal no difference between control and IKE treated group after 48 and 72 hours post dye injection, indicating the ferroptotic cell death is followed by clearance of the dead cells and dye. Mean ± s.d. p-values are reported above the lines n=5 mice (Ordinary one-way ANOVA with Tukey's multiple comparisons). Scalebar 50 mm

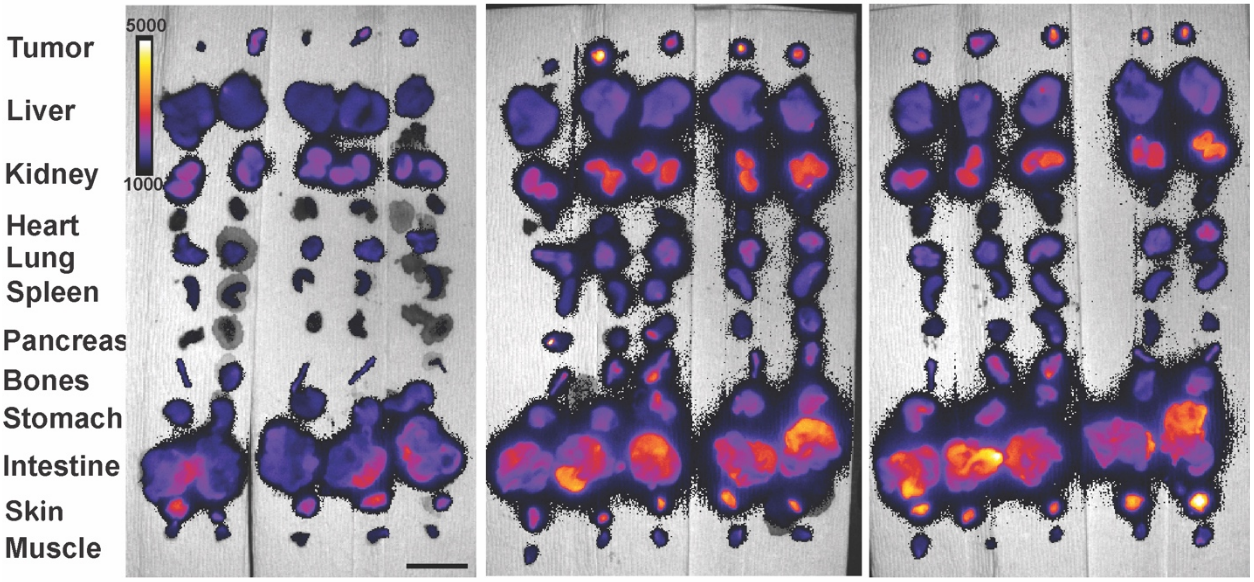

Supplementary Figure 11. Biodistribution of CJ215 in xenograft mice models with and without ferroptosis inducers. Biodistribution image of different organs from MDA MB 435 tumor bearing xenograft mice treated with vehicle(control), IKE and IKE-Liproxstatin, n=5 mice Scalebar 50 mm.

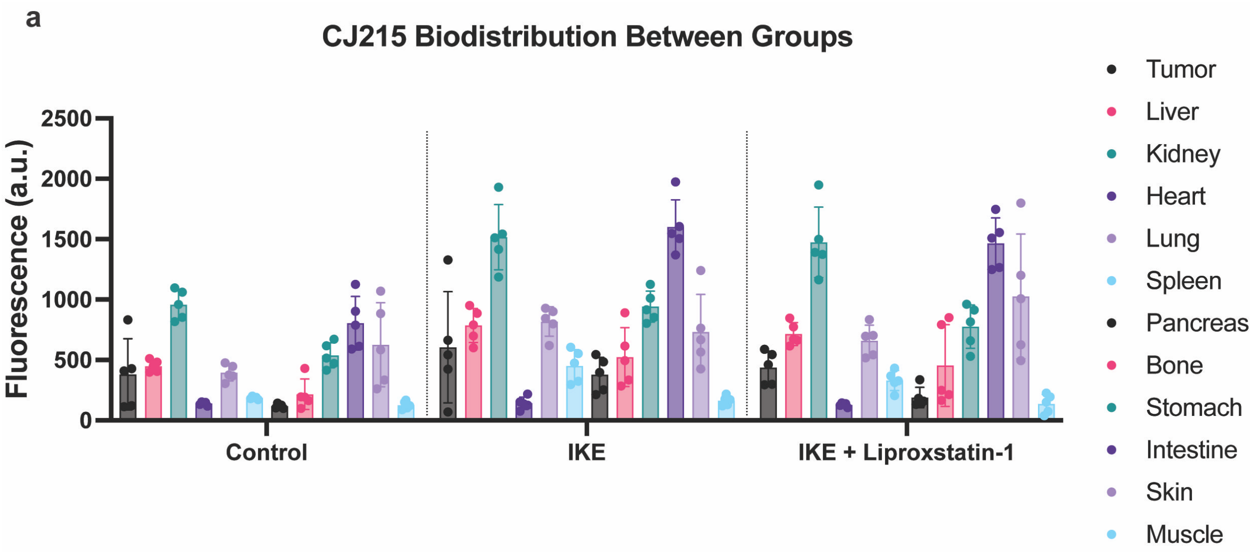

Supplementary Figure 12. Quantifying biodistribution of CJ215 in xenograft mice models with and without ferroptosis inducers in graph. Same graph as Figure 4d reordered and presented in a way that compares organs with one another in the same group with mice groups treated with vehicle(control), IKE and IKE-Liproxstatin indicating off-target effects of IKE treatment in inducing ferroptosis in specific organs,Mean ± s.d. n=5.

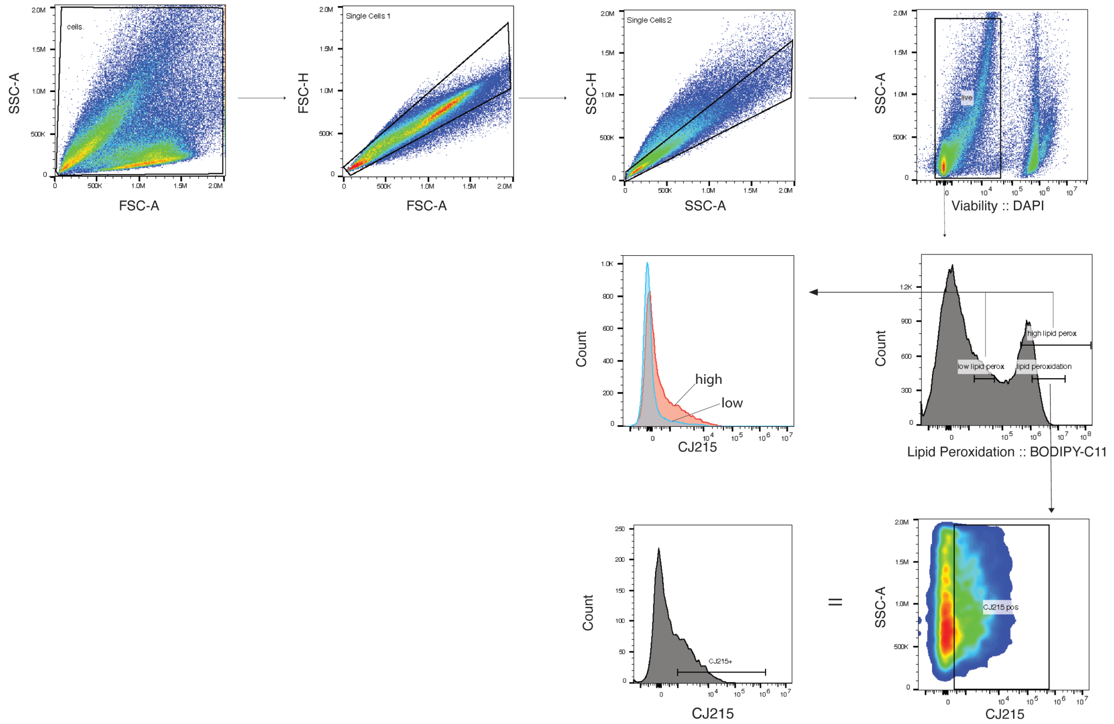

Supplementary Figure 13. Gating Strategy for determining the correlation between Lipid Peroxidation and CJ215. This figure demonstrates the gating steps performed to determine live cell populations and gating high and low lipid peroxidation populations based on BodipyC11 signal along with subsequent determination of CJ215 positive cell populations.

nature portfolio

Corresponding author(s): Jan Grimm

Last updated by author(s): Jul 25, 2025

Reporting Summary

Nature Portfolio wishes to improve the reproducibility of the work that we publish. This form provides structure for consistency and transparency in reporting. For further information on Nature Portfolio policies, see our Editorial Policies and the Editorial Policy Checklist.

Please do not complete any field with "not applicable" or n/a. Refer to the help text for what text to use if an item is not relevant to your study.
For final submission: please carefully check your responses for accuracy; you will not be able to make changes later.

Statistics

For all statistical analyses, confirm that the following items are present in the figure legend, table legend, main text, or Methods section.

n/a	Confirmed	
☐	☒	The exact sample size (n) for each experimental group/condition, given as a discrete number and unit of measurement
☐	☒	A statement on whether measurements were taken from distinct samples or whether the same sample was measured repeatedly
☐	☒	The statistical test(s) used AND whether they are one- or two-sided<br>*Only common tests should be described solely by name; describe more complex techniques in the Methods section.*
☐	☒	A description of all covariates tested
☒	☐	A description of any assumptions or corrections, such as tests of normality and adjustment for multiple comparisons
☐	☒	A full description of the statistical parameters including central tendency (e.g. means) or other basic estimates (e.g. regression coefficient) AND variation (e.g. standard deviation) or associated estimates of uncertainty (e.g. confidence intervals)
☐	☒	For null hypothesis testing, the test statistic (e.g. F, t, r) with confidence intervals, effect sizes, degrees of freedom and P value noted<br>*Give P values as exact values whenever suitable.*
☒	☐	For Bayesian analysis, information on the choice of priors and Markov chain Monte Carlo settings
☒	☐	For hierarchical and complex designs, identification of the appropriate level for tests and full reporting of outcomes
☒	☐	Estimates of effect sizes (e.g. Cohen's d, Pearson's r), indicating how they were calculated

Our web collection on statistics for biologists contains articles on many of the points above.

Software and code

Policy information about availability of computer code

Data collection	Cellular toxicity and mitochondrial membrane potential measurements were done using SpectraMax ID5e(Molecular Devices) utilizing luminescence and fluorescence modes utilizing SpectraMax software. Flow Cytometry assays for lipid peroxidation, quantum dot uptake and annexin V stainings were performed using MACSQuant with 405 nm, 488 nm and 640 nm laser lines and emission detectors FSC: 488/10 nm, SSC: 488/10 nm, V1: 450/50 nm, V2: 525/50 nm, B1: 525/50 nm, B2: 585/40 nm, B3: 655–730 nm, B4: 750 nm LP, R1: 655–730 nm, R2: 750 nm LP and acquired with MACSQuantify software. CJ215 uptake was measured using Odyssey CLx (Li-COR). IVIS system (IVIS Spectrum, Perkin Elmer) in fluorescence mode according to ICG presets (745 excitation, 840 emission, f stop 1, lamp high, small binning). IVIS data were saved by the system automatically, and 'Luminescent' tiff files were used for analysis.
Data analysis	Data analysis for flow cytometry experiments were done with FlowJo version 10 where the cells were gated and their quantifications are exported as raw data. Similarly, images obtained with the imaging experiments with IVIS and Odyssey Systems were exported and analyzed with Fiji for image processing such as background subtraction and obtaining the mean values from various region of interests. These quantifications were later exported as data points. All subsequent statistical analysis were performed by incorporating exported data into Graphpad Prism 9 applying appropriate statistical tests as described in the figure legends of each figure in the main manuscript.

For manuscripts utilizing custom algorithms or software that are central to the research but not yet described in published literature, software must be made available to editors and reviewers. We strongly encourage code deposition in a community repository (e.g. GitHub). See the Nature Portfolio guidelines for submitting code & software for further information.

Data

Policy information about availability of data

All manuscripts must include a data availability statement. This statement should provide the following information, where applicable:

- Accession codes, unique identifiers, or web links for publicly available datasets
- A description of any restrictions on data availability
- For clinical datasets or third party data, please ensure that the statement adheres to our policy

Data is available within the main and supporting figures of the manuscript. Raw data used in the manuscript can be provided for research purposes by the corresponding author depending on the reasoning.

Research involving human participants, their data, or biological material

Policy information about studies with human participants or human data. See also policy information about sex, gender (identity/presentation), and sexual orientation and race, ethnicity and racism.

Reporting on sex and gender	This study did not involve human participants.
Reporting on race, ethnicity, or other socially relevant groupings	-
Population characteristics	-
Recruitment	-
Ethics oversight	-

Note that full information on the approval of the study protocol must also be provided in the manuscript.

Field-specific reporting

Please select the one below that is the best fit for your research. If you are not sure, read the appropriate sections before making your selection.

☒ Life sciences ☐ Behavioural & social sciences ☐ Ecological, evolutionary & environmental sciences

For a reference copy of the document with all sections, see nature.com/documents/nr-reporting-summary-flat.pdf

Life sciences study design

All studies must disclose on these points even when the disclosure is negative.

Sample size	For the in vitro experiments, we performed at least 3 biological replicates and observed the standard deviation between the replications were sufficient to provide enough statistical power for our experiments. For the in vivo experiments, we previously utilized CJ215 and observed its uptake and tumor signal in various conditions and performed additional preliminary experiments, which allowed us to determine using unpaired t-tests that n=5 mice would be sufficient to provide 0.9 statistical power.
Data exclusions	No data was excluded from this study.
Replication	In vitro experiments were replicated with at least 3 biological replicates with multiple technical replicates and routine checks to ensure proper functioning of instruments and the well-being of the cell lines.
Randomization	For the in vitro experiments, no randomization was needed as the cell lines were identical. For the xenograft mice experiments, after the cancer cell injection and tumor growth, the mice were randomized to ensure a consistent distribution across all groups.
Blinding	There was no blinding in the study as animal handling was different between each group and experimenter was responsible for both imaging, injections and analysis at the same time.

Reporting for specific materials, systems and methods

We require information from authors about some types of materials, experimental systems and methods used in many studies. Here, indicate whether each material, system or method listed is relevant to your study. If you are not sure if a list item applies to your research, read the appropriate section before selecting a response.

Materials & experimental systems

n/a	Involved in the study
☒	☐ Antibodies
☐	☒ Eukaryotic cell lines
☒	☐ Palaeontology and archaeology
☐	☒ Animals and other organisms
☒	☐ Clinical data
☒	☐ Dual use research of concern
☒	☐ Plants

Methods

n/a	Involved in the study
☒	☐ ChIP-seq
☐	☒ Flow cytometry
☒	☐ MRI-based neuroimaging

Eukaryotic cell lines

Policy information about cell lines and Sex and Gender in Research

Cell line source(s)	HT-1080: source ATCC(CCL-121)<br>MDA-MD-435: was obtained via Jason Lewis Lab and subsequently authenticated with short tandem repeat analysis.
Authentication	Cell lines were authenticated using Short Tandem Repeat Analysis.
Mycoplasma contamination	The cell lines did not show mycoplasma contamination after the testing.
Commonly misidentified lines (See ICLAC register)	No commonly misidentified lies were used in this study.

Animals and other research organisms

Policy information about studies involving animals; ARRIVE guidelines recommended for reporting animal research, and Sex and Gender in Research

Laboratory animals	In this study, we utilized female mice (Mus musculus) which were from the strain FoxN1nu (nude mice) that were 6 weeks old at the time of the xenograft injections. The mice received food and water and stayed under a 12 hour on/off light cycle with 5 mice per cage.
Wild animals	This study did not involve wild animals.
Reporting on sex	We only used female mice during this study. Given both MDA-MB-435 and HT-1080 cell lines possess androgen receptors their growth profile and migratory behavior would change significantly between sexes. This would influence fatty acid metabolism and CJ215 binding, serving as confounding factors.
Field-collected samples	This study did not involve samples collected from the field.
Ethics oversight	All mouse handling, experimentation, imaging, and housing was conducted according to NIH guidelines that are approved by IACUC protocols. Animal well-being was monitored both by MSKCC's RARC facility and researchers.

Note that full information on the approval of the study protocol must also be provided in the manuscript.

Plants

Seed stocks	No plants were used in this study.
Novel plant genotypes	-
Authentication	-

Flow Cytometry

Plots

Confirm that:

☒ The axis labels state the marker and fluorochrome used (e.g. CD4-FITC).

☒ The axis scales are clearly visible. Include numbers along axes only for bottom left plot of group (a 'group' is an analysis of identical markers).

☒ All plots are contour plots with outliers or pseudocolor plots.

☒ A numerical value for number of cells or percentage (with statistics) is provided.

Methodology

Sample preparation	Samples are prepared as either from the cell lines MDA-MB-435 or HT1080 where cells were cultured in 24 well plates to 80% confluency and treated with the drugs or inhibitors for specified times. Cells were later treated with Lipid Peroxidation dyes, trypsinized and washed with PBS buffer containing 2% FBS twice. After the second wash, cells are incubated with DAPI 1:1000 dilution for 2 minutes and analyzed in the MACS Quant flow cytometer. For the Quantum Dot and Annexin V dataset, the cells were treated with drugs, tyrpsinized, isolated and washed with PBS containing 2% FBS. Afterwards, the cells were incubated either with different quantum dots on ice for 5 minutes to test passive uptake through the pores or 20 minutes with Annexin V per manufacturers instructions. Then the samples were analyzed in MACS Quant System.<br><br>For the ex vivo analysis, kidneys were isolated from mice groups either control, IKE or IKE liproxstatin. Then the kidneys were homogenized and filtered through a 70 um filter, after which red blood cells were lysed. The kidneys were washed with buffer (PBS with 0.5% BSA) and then incubated with BODIPY-C11 for 1 hour at room temperature. After subsequent washing, the samples were stained with 1:1000 dilution of DAPI and read on a CytoFLEX Lx cytometer.
Instrument	This paper utilized flow cytometers MACS Quant10 for the cell culture based analysis along with CytoFLEX Lx cytometer for the ex vivo analysis.
Software	Software used for the data acquisition was MAXQuantify for the MaxQuant system and Cytexpert Software for the CytoFLEX Lx system (Beckman Coulter). After the acqusition, the data were analyzed and gated using FlowJo 10 program.
Cell population abundance	Given this work involved mostly cell culture based flow cytometry assays, our cells were produced in aseptic conditions as monocultures without contamination from other cell lines. In each sample we would have at least around 30.000 cells after the staining and washing steps. For the ex vivo analysis, we isolated kidneys from the animals, homogenized and passed it through cell strainers to ensure sterility of our samples. As we wanted to capture overall lipid peroxidation in all the cells, we did not perform sorting and we analyzed more than 10.000 cells in each sample.
Gating strategy	As this study looks at all the cell populations within the kidney, we did not distinguish specific populations within the forward to side scattered plot, but we did select for singlets in both forward scatter and side scatter plots separately. Then we gated for live cell populations and measured Bodipy-C11 signal. Then we selected low and high lipid peroxidation populations based on 10% of the populations in either side of the second peak that shows Lipid Peroxidation signal within the graph and measured their mean fluorescence intensity in CJ215 channel. We also looked at the highest lipid peroxidation populations and observed an increase in CJ215 uptake, which we dubbed as CJ215+ population.

☒ Tick this box to confirm that a figure exemplifying the gating strategy is provided in the Supplementary Information.

This checklist template is licensed under a Creative Commons Attribution 4.0 International License, which permits use, sharing, adaptation, distribution and reproduction in any medium or format, as long as you give appropriate credit to the original author(s) and the source, provide a link to the Creative Commons license, and indicate if changes were made. The images or other third party material in this article are included in the article's Creative Commons license, unless indicated otherwise in a credit line to the material. If material is not included in the article's Creative Commons license and your intended use is not permitted by statutory regulation or exceeds the permitted use, you will need to obtain permission directly from the copyright holder. To view a copy of this license, visit http://creativecommons.org/licenses/by/4.0/
